# Efficacy and safety of manufactured Chinese herbal formula for cervical radiculopathy: a systematic review with meta-analysis and trial sequential analysis

**DOI:** 10.3389/fnins.2026.1721569

**Published:** 2026-02-10

**Authors:** Bin Tang, Xiaohang Bao, Canmei Li, Weixiong Gan, Wei Xu, Yisheng Zhang, Zhuotan Wu, Zhifei Li, Baohua Huang

**Affiliations:** 1 The First Affiliated Hospital of Guangxi University of Chinese Medicine, Guangxi, Nanning, China; 2 Guangxi University of Chinese Medicine, Guangxi, Nanning, China

**Keywords:** cervical radiculopathy, efficacy and safety, manufactured Chinese herbal formula, meta-analysis, trial sequential analysis

## Abstract

**Background:**

Clinical trials have been widely reported for the application of manufactured Chinese herbal formula (MCHF) in patients with Cervical Radiculopathy (CR). Existing published systematic reviews are confined to assessing the therapeutic efficacy of individual MCHF types in the management of CR, whereas a comprehensive, holistic evaluation of both efficacy and safety outcomes across the full spectrum of MCHF remains lacking.

**Objective:**

To assess the efficacy and safety of MCHF for CR.

**Methods:**

We searched six databases and three registries from inception to 30 September 2024. Eligible randomized controlled trials investigating the efficacy of MCHF for CR were included. Data extraction, methodological assessment, and meta-analyses were conducted according to the Cochrane standards.

**Results:**

We included 14 studies, covering 8 types of MCHF with a total of 1,576 participants. MCHF may reduce the pain of VAS in CR. In subgroup meta-analysis, MCHF may be more efficacious in relieving pain than the placebo. MCHF combined with conventional oral drugs were more efficacious than conventional oral drugs alone. Trial sequential analysis (TSA) indicated that the sample sizes were adequate to confirm the reliability of the aforementioned findings. No significant difference in therapeutic efficacy was observed between MCHF and conventional oral medications, but TSA indicated that further large-scale RCTs are required to validate these results. In terms of the Neck Disability Index (NDI), the intervention group showed potentially superior functional improvement compared with the control group in add-on and placebo-controlled trials; however, its efficacy relative to conventional oral drugs remained unclear. Regarding adverse events, no significant between-group differences were detected in add-on and head-to-head trials, whereas the intervention group had a higher incidence of adverse events than the control group in placebo-controlled trials.

**Conclusion:**

MCHF may be efficacious in the pain and neck disability in treatment of CR, and may not exhibit significant differences from conventional oral drugs. A certain incidence of adverse events was observed in MCHF-treated groups. However, the certainty of evidence is low/very low, with safety evidence being particularly weak. Further high-quality RCTs are warranted to verify the efficacy and safety of MCHF.

**Systematic review registration:**

[https://www.crd.york.ac.uk/PROSPERO/view/CRD42024605787], identifier [#CRD42024605787].

## 1 Background

Cervical spondylosis is one of the most common musculoskeletal disorders, with approximately 30–50% of adults experiencing neck pain each year, and an average annual incidence rate of 37.2% (Carroll et al., 2008; Cohen, 2015; Fejer et al., 2006). The age-standardized global prevalence and incidence of cervical spondylosis are 3.55% and 0.81%, respectively (Safiri et al., 2020). The healthcare expenditure and disability burden of cervical and lumbar spine-related diseases are the highest among all diseases globally (Kazeminasab et al., 2022). Cervical radiculopathy (CR), also referred to as cervical spondylotic radiculopathy (CSR), is the most prevalent subtype of cervical spondylosis, accounting for approximately 60–70% of all patients (Bono et al., 2011). CR is caused by compression or stimulation of the dorsal sensory and ventral motor roots of the cervical nerves at one or more vertebral segments, resulting in corresponding motor and sensory dysfunction (McCartney et al., 2018). It may manifest as dizziness, headache, tinnitus, gastrointestinal pain, hypomnesia, palpitations, and upper arm neuropathy (Leung et al., 2023). Most CR patients are young and middle-aged individuals who are highly productive, especially in the age group of 50–60 years (Rainville et al., 2019). The cost of treatment, reduced productivity, and other issues caused by the disease have led to a significant economic burden on society.

In clinical practice, most patients are treated with non-surgical strategies. Non-steroidal anti-inflammatory drugs (NSAIDs) and opioid receptor analgesics are widely used in different regions, but they have side effects such as gastrointestinal reactions and addiction (Childress and Becker, 2016). Alternative medicine such as spinal manipulation, accupunturem and Chinese herbal medicinel are widely used in management of CR and the Chinese herbal medicine offers significant advantages and characteristics in the management (Chu et al., 2023).

According to Traditional Chinese Medicine (TCM) theory, CR is characterized by either “Qi stagnation and blood stasis syndrome” or “deficiency of liver-yin and kidney-yin syndrome.” Over time, these imbalances lead to “meridian obstruction,” which ultimately results in pain (Xue and Liu, 2025).

Medicinal Chinese herbal formulations (MCHF), also known as Chinese patent medicines (CPM), are regulatory-approved standardized herbal preparations that represent a unique class of botanical medicinal products. These formulations integrate multiple medicinal plants based on evidence-based compatibility principles to optimize therapeutic outcomes through synergistic interactions, while minimizing potential adverse effects (Li et al., 2024). More than 200 MCHF formulations have received certification from the National Medical Products Administration (NMPA) under Good Manufacturing Practice (GMP) standards. Notably, 63% of these formulations are included in the National Essential Drug List (2012 Edition), serving as fundamental therapies within China’s primary healthcare system.

MCHF have been widely used in the treatment of CR and related diseases across various clinical departments, demonstrating significant therapeutic efficacy and good safety profiles. In contrast, traditional crude herbal decoctions face several challenges in basic research, including variability in the quality of medicinal materials and poor reproducibility between batches. MCHFs, however, are produced following rigorous preparation processes and quality control standards, ensuring the stability of their compound composition for research purposes. These standardized formulations are extensively used in China and are increasingly gaining international recognition (Yao et al., 2015). Therefore, the systematic evaluation of Chinese patent medicines is well-supported by evidence of their clinical efficacy, providing a strong foundation for the wider adoption of these treatments.

In recent years, numerous clinical trials investigating the use of MCHF in patients with CR have been published in both English and Chinese medical journals (Zhu et al., 2015a). While several systematic reviews and meta-analyses have focused on specific formulations of MCHF for CR, these studies are limited by their narrow scope and thus cannot provide comprehensive evidence for the overall efficacy and safety of MCHF as a therapeutic class in the management of CR. The objective of this systematic review and meta-analysis is to comprehensively evaluate the efficacy and safety profiles of different MCHF for the treatment of CR, through a rigorous synthesis of data from all relevant published RCTs. Furthermore, we performed subgroup analyses for placebo-controlled trials, add-on trials, and head-to-head trials, as well as subgroup and sensitivity analyses for each individual MCHF formulation, to enhance the relevance of our findings to real-world clinical practice settings.

## 2 Method

### 2.1 Protocol registration

This study protocol has been registered in PROSPERO.^1^ It adheres to the PRISMA-P guidelines for systematic review protocols, while the subsequent systematic review and meta-analysis follow the PRISMA standards (Liberati et al., 2009; Shamseer et al., 2015).

### 2.2 Study objective

The aim of this review was to explore the efficacy and safety of MCHF in the treatment of CR.

### 2.3 PICOs

#### 2.3.1 Participant

We included participant that confirmed diagnosis of CR with corresponding signs and symptoms of radicular nerve compression or irritation (Bono et al., 2011).

#### 2.3.2 Interventions

Studies in which MCHF was compared with placebo, conventional oral drug, or the combination of MCHF and conventional oral drug vs. conventional oral drug alone, regardless of dose or treatment duration. MCHFs refer to Chinese patent medicines approved by the NMPA.

#### 2.3.3 Comparator

Control groups included placebo, conventional oral drug alone, or in combination. Conventional medications are as follows:

1. Non-steroidal anti-inflammatory drugs (NSAIDs): e.g., Celecoxib Capsules, Aceclofenac Sodium capsules.
2. Muscle relaxants: e.g., Tizanidine Hydrochloride tablets, Eperisone Hydrochloride tablets.
3. Analgesics: e.g., Loxoprofen Sodium and Codeine Sustained-release tablets.
4. Neurotrophic agents: e.g., Mecobalamin tablets, Neroxon tablets.

#### 2.3.4 Outcomes

The primary outcome was the Visual Analog Scale (VAS) for global pain at the end of treatment. Secondary outcomes included the Japanese Orthopedic Association (JOA) Score, Neck Disability Index (NDI), and the 36-Item Short Form Health Survey (SF-36) assessed at the end of treatment, as well as the incidence of adverse events during treatment (Liang et al., 2019; Rajjoub et al., 2024; Zhu et al., 2015a).

#### 2.3.5 Study type

We had included randomized controlled trial (RCT) to assess the beneficial efficacy and safety of the treatments.

### 2.4 Exclusion criteria

We used the following exclusion criteria: (a) Inclusion of MCHF in both the intervention and control groups; (b) Combination therapy including herbal medicine, acupuncture, moxibustion, manual therapy, tai chi, and yoga were excluded. (c) Reviews, animal experiments and conference abstracts; (d) Studies that presented insufficient data or duplicate publications.

### 2.5 Database and search strategies

Six databases of PubMed, Excerpta Medica Database (EMBASE), Cochrane Central Register of Controlled Trials (CENTRAL), National Knowledge Infrastructure (CNKI), Wanfang Digital Periodicals (WANFANG), Chinese Scientific Journal Database (VIP), was searched systematically to identify RCTs of MCHF for patients with CR. Additionally, a supplementary search for ongoing trials was performed in three international clinical trial registries: ClinicalTrials.gov,^2^ the EU Clinical Trials Register and Clinical Trials Information System (CTIS),^3^ and the World Health Organization (WHO) International Clinical Trials Registry Platform (ICTRP).^4^ The search strategy was pre-registered in October 2024, and all relevant studies were identified by electronic database search from inception until September 30, 2024. The following search terms are used individually or in combination: “cervical spondylotic radiculopathy”; “cervical radiculopathy”; “nerve root type cervical spondylosis”; “Chinese”; “tablet”; “pill”; “powder”; “capsule”; “granule”; “oralliquid.” The detailed search terms are displayed in Supplementary Material 1. There were no restrictions on language. The searches had been re-run prior to the final analysis.

### 2.6 Citation management and screening

All records underwent automated deduplication using NoteExpress v 4.0. Two authors (Bin Tang and Xiaohang Bao) independently screened the titles and abstracts based on predefined inclusion and exclusion criteria for preliminary selection. When titles and abstracts provided insufficient information, full-text articles were reviewed by the same two researchers. Any disagreements were resolved through discussion, with final consensus achieved by involving other reviewers (Baohua Huang).

### 2.7 Data extraction

Two reviewers conducted data extraction (Bin Tang and Xiaohang Bao) separately according to the preset contents. Disagreement was resolved by discussion and reached consensus through a third reviewer (Baohua Huang). The extracted data included first author names, year of publication, sample size, population characteristics (age and sex of patients), intervention, duration of treatment, all study outcome assessment (VAS, JOA, NDI, SF-36, and adverse events), and overall conclusions about the efficacy of MCHF. Intervention and control group details included the name of the drug, dosage, therapeutic regimen, treatment duration. We extracted the outcome data after the last treatment. If the outcome data were reported in other formats, they were converted to means and standard deviations (SDs).

### 2.8 Risk of bias

The methodological quality of the included studies was independently assessed by Bin Tang and Xiaohang Bao using the Risk of Bias 2 (RoB 2) tool, following the criteria outlined in the Cochrane Handbook for Systematic Reviews of Interventions (Higgins et al., 2019). The following domains were evaluated: bias arising from the randomization process, bias due to deviations from intended interventions, bias due to missing outcome data, bias in measurement of the outcome, and bias in selection of the reported result (Sterne et al., 2019). We used the Shiny web application as a visualization tool for generating risk of bias (RoB2) figures specific to randomized controlled trials (McGuinness and Higgins, 2021). Any discrepancies in the risk of bias assessments were resolved through discussion or consultation with a third reviewer (Wei Xu).

### 2.9 Data synthesis

We used Review Manager software (RevMan, version 5.4; Cochrane Collaboration) to conduct the data analysis. The minimum number of studies required for meta-analysis was two (Myung, 2023). Continuous outcomes were reported as mean differences (MD) or standardized mean differences (SMD) with 95% confidence intervals (CI). Binary outcomes were reported as risk ratios (RR) or odds ratios (OR), also with 95% CI. Heterogeneity among studies was assessed using the I^2^ statistic. If substantial heterogeneity was detected (*I*^2^ > 50%), a random-effects model was used to calculate the pooled estimates; otherwise, a fixed-effect model was applied. If the included studies are not suitable for meta-analysis, this will be explicitly stated and explained in the results section (Higgins et al., 2024). For rare events or zero-cell study events, the Peto odds ratio (Peto OR) or continuity correction method was employed to re-pool the effect sizes. If the included studies are not suitable for meta-analysis, this will be explicitly stated and explained in the results section.

### 2.10 Subgroup analysis

We conducted subgroup analyses for each comparison type, including MCHF vs. placebo, MCHF vs. conventional oral drug, and MCHF combined with conventional oral drug vs. conventional oral drug alone. Based on the specific names of MCHF in the same type of comparison, we conducted further subgroup analysis on the primary outcome measures. Potential sources of heterogeneity were explored through additional subgroup analyses, including publication year, randomization method, sample size, gender ratio, and treatment duration. We also calculated *p*-values for interaction (P_*interaction*_) to evaluate the differences between different subgroups.

### 2.11 Sensitivity analyses

Sensitivity analyses were focused on the primary outcome to assess the robustness of the results, using approaches such as changing the statistical model (fixed-effect vs. random-effects) and excluding specific types of studies. If the direction of the results remains unchanged, it indicates that the results are stable; if the direction changes, the results are deemed unstable.

### 2.12 Assessment of publication biases

We had visualized funnel plots using Statistics and Data Analysis (Stata, version 15.0) to assess publication bias (funnel threshold ≥ 10 studies). A qualitative assessment was performed by visual inspection of funnel plot symmetry, while a quantitative evaluation was conducted using Egger’s test and Begg’s test. When publication bias was suggested, the Duval and Tweedie trim-and-fill method was applied to estimate the number of potentially missing studies and to assess the impact of publication bias on the pooled effect size (Duval and Tweedie, 2000). Meta-regression analyses in the present study were performed using Stata 15.0 software, with the “metareg” command employed to fit a random-effects model. This model was used to evaluate whether the duration of treatment exerted an impact on VAS outcomes. Collinearity checks were performed before analysis, and statistical significance was determined at *P* < 0.05.

### 2.13 Qualitative analysis of evidence level

We used the Grading of Recommendations Assessment, Development, and Evaluation (GRADE) approach to assess the quality of evidence for both primary and secondary outcome measures in the meta-analysis. GRADE evaluated five domains: risk of bias, indirectness, imprecision, inconsistency, and publication bias (Higgins et al., 2019; Rajjoub et al., 2024). The assessment was conducted using GRADEpro software (version 3.6.1; available at: gradepro.org).

### 2.14 Trial sequential analysis

Trial sequential analysis (TSA) was performed to the primary efficacy measure. Trial sequential analysis (TSA) was conducted with the Copenhagen Trial Unit’s dedicated software (v0.9.5.10 beta, 2021 release) to determine the conclusiveness of current evidence and guide future investigation design. The analysis incorporated three key components: (1) Calculation of heterogeneity-adjusted required information size (RIS) accounting for both between-study variance (DerSimonian-Laird estimator) and cumulative random error. (2) Construction of monitoring boundaries using the O’Brien-Fleming α-spending function. (3) Evaluation of accrued information against predefined thresholds (α = 5%, power = 80%). This methodology enabled a quantitative assessment of the reliability of meta-analytic conclusions. If the cumulative Z-curve crosses the trial sequential monitoring boundary, it indicates that firm evidence has been reached. If it crosses the futility boundary adjusted for the RIS, it suggests that further trials are unlikely to change the conclusion. However, if the Z-curve remains within the area of uncertainty, additional research is needed (Brok et al., 2008; Watterslev et al., 2008). In theory, robust evidence can be considered established when the Z-curve crosses either the monitoring boundary or reaches the required information size. Conversely, if neither is crossed, additional trials are likely needed to reach conclusive evidence.

**TABLE 1:** General information of the articles included in this review.

Study ID	Sample size	Age	Sex (male/female)	Diagnostic criteria	Intervention		Duration of treatment (days)	Usage instructions	Randomization method	Outcomes	Adverse events (cases)
					Experimental group	Control group					
Zhang et al. (2024)	EG 10 CG 11	EG 44.78 + 8. 72 CG 45.34 + 7.69	EG 12/20 CG 10/22	Chinese Medical Association (2018)	Shujin Tongluo Granules + Methylcobalamin Tablets	Methylcobalamin Tablets	28	Shujin Tongluo Granules (12 g,tid) Methylcobalamin Tablets (0.5 mg,tid)	Random number table method	VAS;JOA	EG:1
Huang et al. (2023)	EG 41 CG 41	EG 32.26 + 9.17 CG 32.84 + 9.03	EG 24/17 CG 28/13	Chinese Medical Association (2015)	Jingtong Granules + Tizanidine Hydrochloride Tablets	Tizanidine Hydrochloride Tablets	42	Jingtong Granules (4 g,tid) Tizanidine Hydrochloride Tablet (2 mg,tid)	Random number table method	VAS;NDI	EG:4 CG:3
Fu et al. (2023)	EG 30 CG 30	EG 52.50 + 1. 61 CG 51.51 + 1.34	EG 16/14 CG 18/12	Chinese Medical Association (2015)	Jingfukang Granules + Neurotropin	Neurotropin	14	Jingfukang Granules (5 g,bid) Neurotropin (16.0, bid)	Random number table method	VAS;NDI	EG:3 CG:5
Zhang et al. (2021)	EG 43 CG 43	EG 48.86 + 5.16 CG 49.05 + 5.38	EG 22/21 CG 23/20	Unspecified	Jingtong Granu1es + Compound Codeine Phosphate and Ibuprofen Sustained Release Tablets	Compound Codeine Phosphate and Ibuprofen Sustained Release Tablets	EG:14 CG:5	Jingtong Granules (4 g,qd) Compound Codeine Phosphate and Ibuprofen Sustained Release Tablets (2 tablets, bid)	Unspecified	VAS;NDI	Unspecified
Hu et al. (2020)	EG 359 CG 120	EG47.98 + 10.63 CG48.13 + 10.83	EG 132/227 CG 42/78	Unspecified	Jingshu Granules	Placebo	28	Jingshu Granules (6 g, tid) Placebo (6 g, tid)	SAS	VAS	EG:71 CG:9
Yang et al. (2020)	EG 48 CG 48	EG 47.0 + 11.3 CG 47.0 + 11.1	EG 32/16 CG 28/20	The People’s Republic of China on Traditional Chinese Medicine industry standards (2012)	Jingshu Granu1es + Ce1ecoxib + Eperisone Hydrochloride	Celecoxib + Eperisone Hydrochloride	28	Jingshu Granules (6 g, tid) Celecoxib (0.2 g, qd) Eperisone Hydrochloride (50 mg, bid)	Random number table method	VAS;NDI	Unspecified
Yuan and Li (2017)	EG 41 CG 41	EG 43.63 + 5.91 CG 41.94 + 6.03	EG 18/23 CG 20/21	Chinese Medical Association (2008)	Biqi Capsules	Aceclofenac Capsules	14	Biqi Capsules (1.2 g, bid) Aceclofenac Capsules (0.1 g, bid)	Random number table method	VAS	EG:2 CG:4
Zou and Guo (2017)	EG 64 CG 64	EG 42.13 + 0.83 CG 40.22 + 0.53	EG 41/23 CG 41/23	Chinese Medical Association (1993)	Tenghuang Jiangu Tablets	Celecoxib + Mecobalamin capsules	21	Tenghuang Jiangu Tablets (2 g, tid) Celecoxib (200 mg, QD) Mecobalamin capsules (500 ug, tid)	Unspecified	VAS	None
Zhang (2015)	EG 23 CG 23	Unspecified	Unspecified	Chinese Medical Association (1993)	Qishe pill	Placebo	28	Qishe pill (3.75 g, bid) Placebo (3.75 g, bid)	SAS	VAS;NDI	CG:1
Shi et al. (2014)	EG 48 CG 48	EG 48.98 + 5.82 CG 50.12 + 6.47	EG 28/20 CG 25/23	The People’s Republic of China on Traditional Chinese Medicine industry standards (2012)	Gentongping Granules	Eperisone	84	Gentongping Granules (12 g, bid) Eperisone (50 mg, tid)	Random number table method	VAS	EG:6 CG: 12
Huang (2014)	EG 24 CG 24	EG 50.8 + 12.08 CG 51.58 + 12.43	EG 7/17 CG 8/16	Chinese Medical Association (1993)	Qishe pill	Placebo	28	Qishe pill (3.75 g, bid) Placebo (3.75 g, bid)	SAS	VAS;NDI;SF36	EG:4 CG:1
Liu et al. (2013)	EG 80 CG 80	Unspecified	EG 43/37 CG 45/35	Chinese Medical Association (1993)	Jingtong Granu1es + Compound Codeine Phosphate and Ibuprofen Sustained Release Tablets	Compound Codeine Phosphate and Ibuprofen Sustained Release Tablets	EG:14 CG:5	Jingtong Granules (1, qd, 14d) Compound Codeine Phosphate and Ibuprofen Sustained Release Tablets (2 tablets, bid, 5d)	Unspecified	VAS;NDI	Unspecified
Huang (2011)	EG 59 CG 59	Unspecified	EG 39/20 CG 35/24	Diagnostic and therapeutic standards for traditional Chinese medicine diseases and syndromes (1944)	Jingtong Granules	Placebo	30	Jingtong Granules (4 g, qd) Placebo (300 mg, qd)	Unspecified	VAS	Unspecified
Liu and Zhang (2008)	EG 84 CG 36	48 + 14	67/53	Unspecified	Jingtong Granules	Placebo	28	Jingtong Granules (4 g, tid) Placebo (300 mg, tid)	Unspecified	VAS	Unspecified

## 3 Results

### 3.1 Retrieval results of literature

**FIGURE 1:**
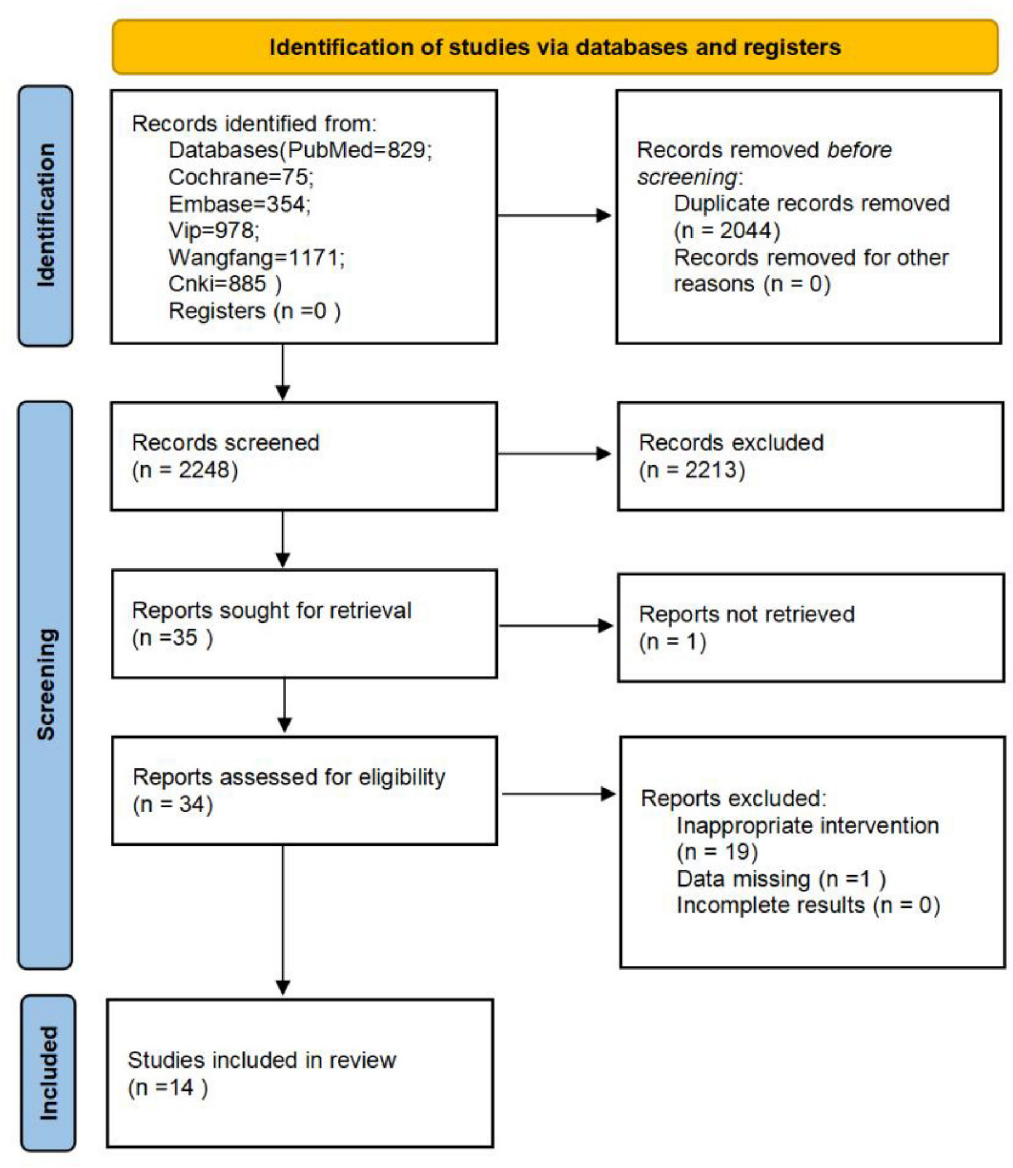
PRISMA 2022 flow diagram.

A total of 4292 articles were identified through the electronic database search, with 2044 duplicates excluded. After screening titles and abstracts, 2,214 articles were further excluded. Additionally, 19 trials were excluded due to inappropriate interventions or outcomes, and 1 trial was excluded due to missing data upon full-text review. Ultimately, 14 randomized controlled trials (RCTs) (Fu et al., 2023; Hu et al., 2020; Huang et al., 2023; Huang, 2011; Huang, 2014; Liu and Zhang, 2008; Liu et al., 2013; Shi et al., 2014; Yang et al., 2020; Yuan and Li, 2017; Zhang et al., 2021; Zhang et al., 2024; Zhang, 2015; Zou and Guo, 2017) met the inclusion criteria and were included in the systematic review and meta-analysis (Table 1). A PRISMA flowchart outlining the study selection process is shown in Figure 1.

### 3.2 Basic characteristics of the included literature

The included randomized controlled trials (RCTs) were published between 2008 and 2024, with a total of 1,576 participants. Four studies (Fu et al., 2023; Shi et al., 2014; Yuan and Li, 2017; Zou and Guo, 2017) employed a head-to-head design, comparing the efficacy of MCHF alone with conventional oral drugs alone. Five studies (Hu et al., 2020; Huang, 2011; Huang, 2014; Liu and Zhang, 2008; Zhang, 2015) compared MCHF with placebo to assess the absolute efficacy of MCHF. The remaining five studies (Huang et al., 2023; Liu et al., 2013; Yang et al., 2020; Zhang et al., 2021; Zhang et al., 2024) utilized a loading study design, comparing the efficacy of MCHF combined with conventional oral drugs to conventional oral drugs alone.

Nine studies were published within the last 10 years, while the remaining five studies were published more than 10 years ago. Two studies had sample sizes of less than 60 participants. The random number table method was employed in six studies, the SAS method was used in three studies, and the methodology for the remaining studies was not specified.

All 14 studies reported changes in VAS, 7 studies reported NDI, 9 studies reported the number of adverse events and 1 study reported SF-36.

### 3.3 Risk bias

**FIGURE 2:**
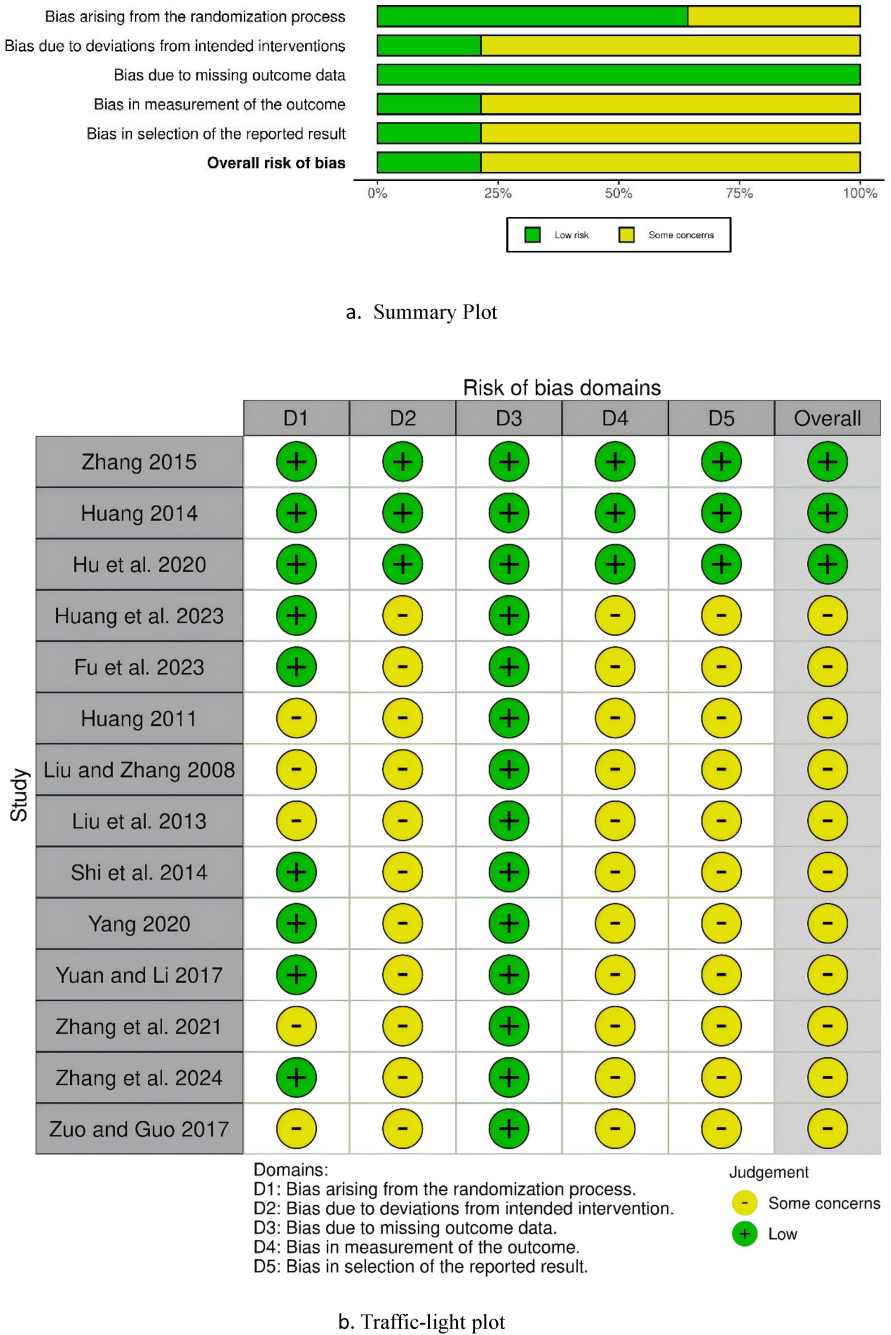
Risk bias assessment. **(a)** Summary Plot. **(b)** Traffic-light plot.

The methodological quality of the included studies was assessed in accordance with the criteria outlined in the Risk of Bias 2 (RoB 2) tool from The Cochrane Handbook for Systematic Reviews of Interventions. Overall, 11 studies were judged to have “Some concerns” regarding overall bias, while 3 studies were rated as having “Low” overall bias. Although all trials reported the use of randomization, only 9 specified the concrete methods employed—such as drawing lots, computer-generated random sequences, stratified randomization, or random number tables. Only 3 studies mentioned allocation concealment, and these 3 studies also implemented double-blinding. Most trials provided no information regarding blinding procedures. Fourteen studies reported no baseline differences between the intervention group and the control group. For three studies, the risk of bias arising from three domains—bias due to deviations from intended interventions, bias in measurement of the outcome, and bias in selection of the reported result—was rated as “Low”; the remaining 11 studies were judged to have “Some concerns” in these domains. All 14 studies were rated as having “Low” risk of bias related to bias due to missing outcome data. The risk of bias assessment plot is shown in Figure 2.

### 3.4 VAS-meta

#### 3.4.1 MCHF vs. placebo and MCHF plus conventional oral drugs vs. conventional oral drugs

**FIGURE 3:**
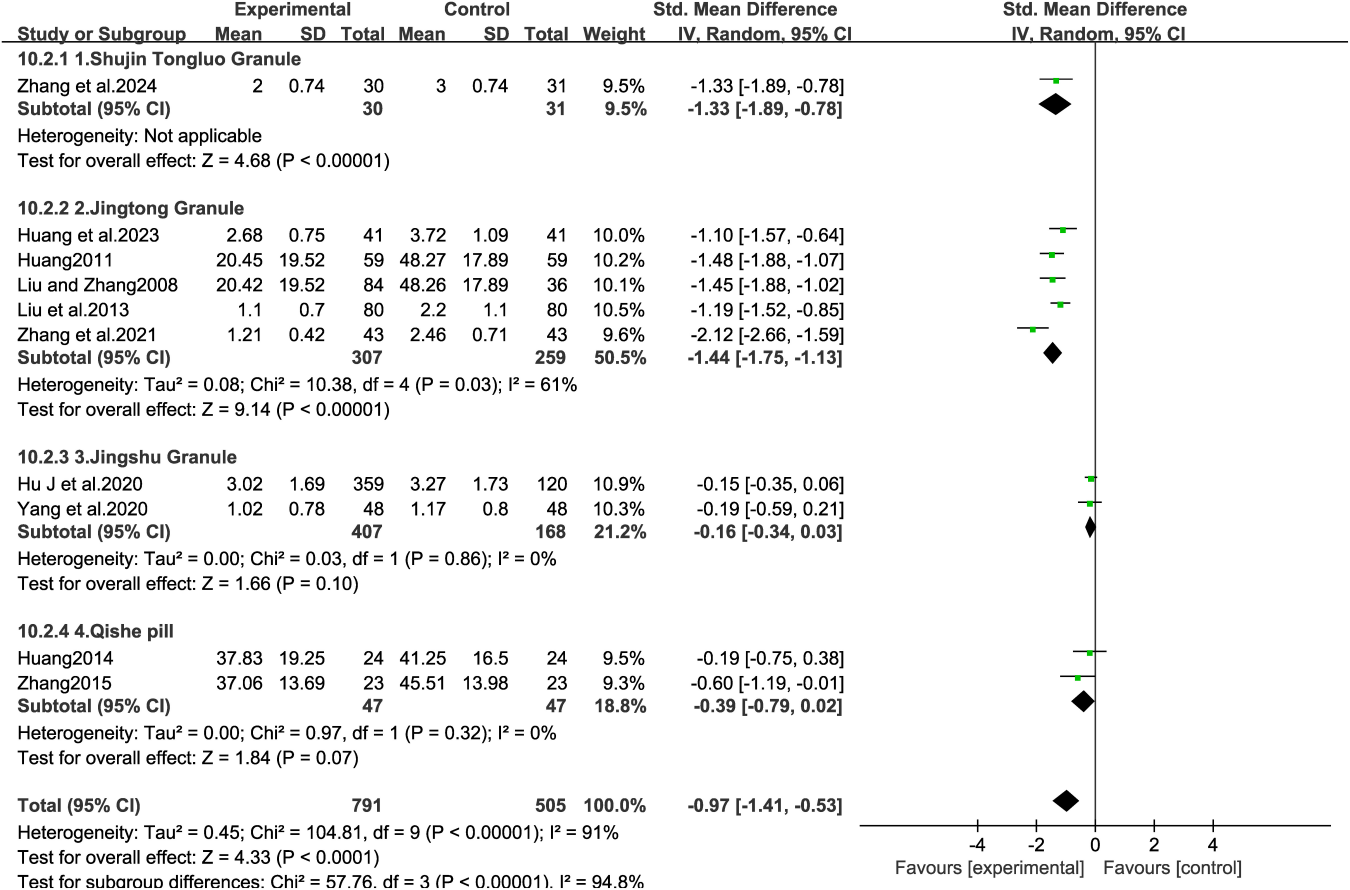
VAS forest plot of MCHF plus conventional oral drugs vs. conventional oral drugs and MCHF vs. placebo.

**FIGURE 4:**
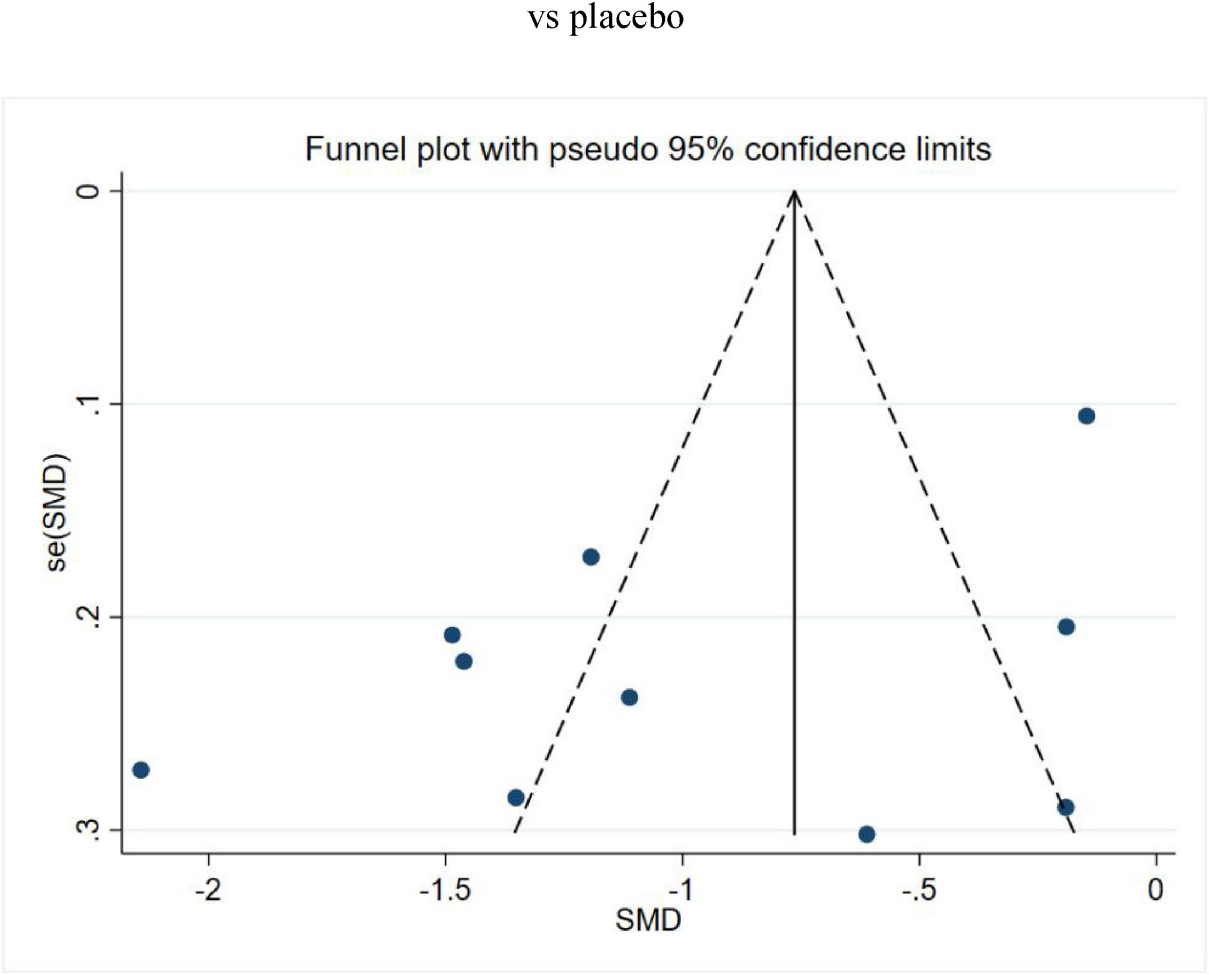
Funnel plot of MCHF plus conventional oral drugs vs. conventional oral drugs and MCHF vs. placebo on VAS.

Ten studies compared the VAS outcomes of MCHF plus conventional oral drugs vs. conventional oral drugs alone, or MCHF vs. placebo (Figure 3). The statistical analysis revealed substantial heterogeneity (*P* = 0.00, *I*^2^ = 91%). Therefore, a random-effects model was applied for the meta-analysis. The results indicated that the MCHF group showed superior therapeutic effects compared to the control group (SMD = -0.97, 95% CI: −1.41 to −0.53, *P* < 0.05). Funnel plot analysis demonstrated approximate symmetry (Figure 4). Both the Egger test (*P* = 0.07) and the Begg test (*P* = 0.86) did not detect significant publication bias.

**FIGURE 5:**
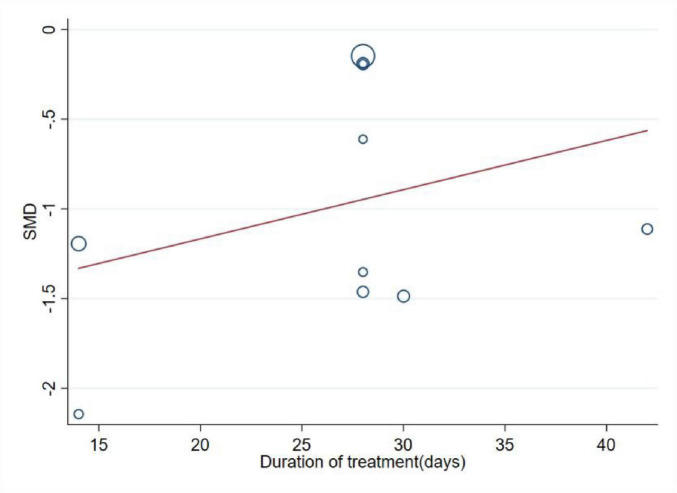
Meta-regression of the effect of duration for MCHF plus conventional oral drugs vs. conventional oral drugs and MCHF vs. placebo on VAS.

Meta-regression revealed no statistically significant association between the duration of MCHF treatment and clinical outcomes in CR (*P* = 0.36) (Figure 5).

Given that the *p*-values of both Egger’s test and Begg’s test were greater than 0.05 and the funnel plots were essentially symmetrical, no significant publication bias was detected, and the trim-and-fill method was therefore not performed.

Although the number of included studies just met the minimum threshold of 10, the overall sample size of the trials remained relatively small. This limitation compromised the statistical power of the meta-regression, funnel plot analysis, and Egger/Begg tests, potentially leading to misleading results.

##### 3.4.1.1 MCHF and placebo

**FIGURE 6:**
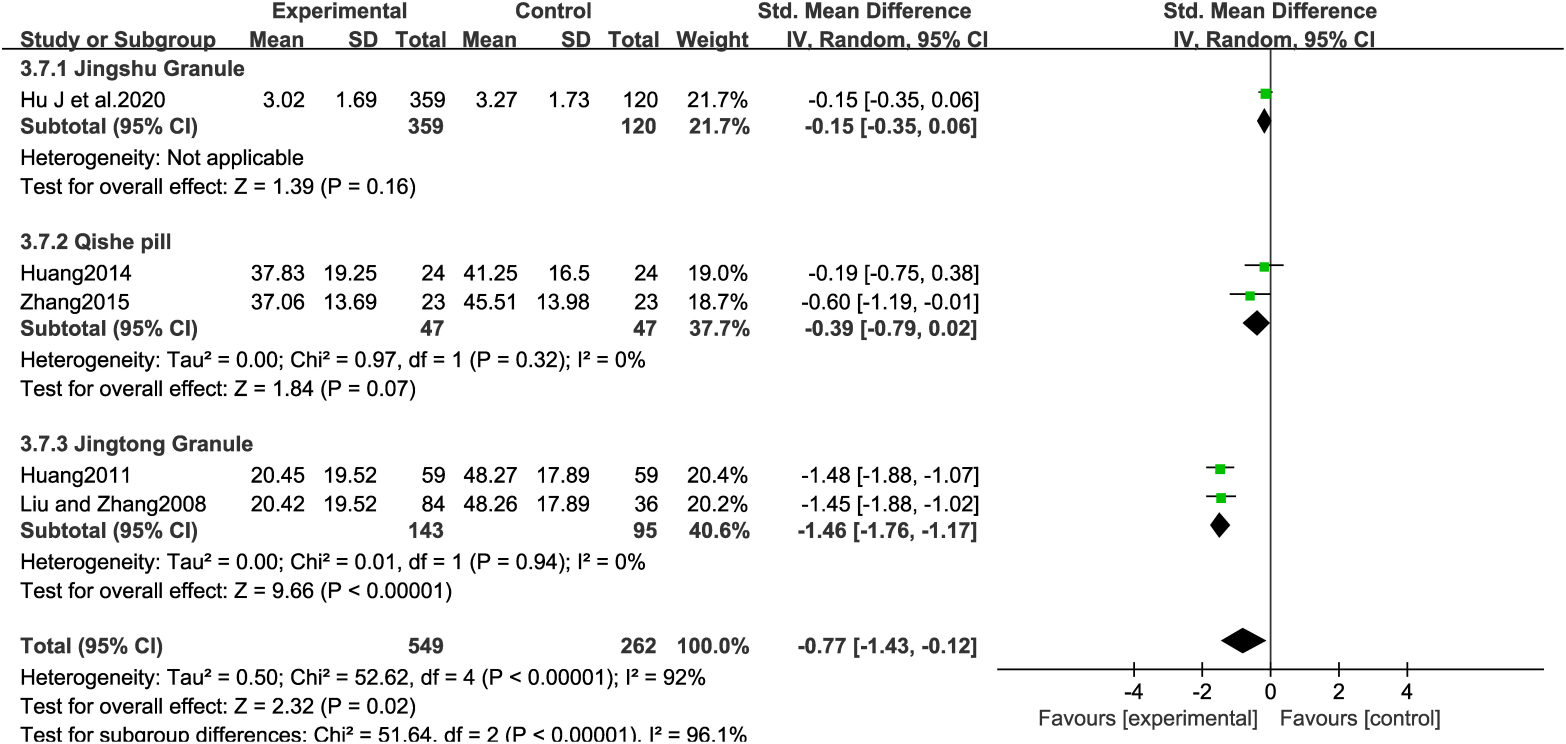
VAS forest plot of MCHF and placebo.

The five included studies (Hu et al., 2020; Huang, 2011; Huang, 2014; Liu and Zhang, 2008; Zhang, 2015), involving 811 patients, compared the efficacy of MCHF with placebo (Figure 6). Pain improvement in the MCHF group was significantly greater than in the placebo group (SMD = -0.77, 95% CI: −1.43 to −0.12, *P* < 0.05; *I*^2^ = 92%). Subgroup analysis revealed that the Jingshu Granule group (Hu et al., 2020) did not show superior efficacy compared to the placebo group. The Qishe Pill group, which included two studies (Huang, 2014; Zhang, 2015), did not show better pain improvement than the placebo (SMD = -0.39, 95% CI: −0.79 to 0.02, *P* = 0.07; *I*^2^ = 0.0%). Similarly, the Jingtong Granule group, consisting of two studies (Huang, 2011; Liu and Zhang, 2008), showed greater pain improvement compared to placebo (SMD = -1.46, 95% CI: −1.76 to −1.17, *P* < 0.05; *I*^2^ = 0%). Both intervention groups exhibited superior efficacy compared to the control group, with significant effect sizes. These findings suggest that the specific type of MCHF plays a significant role in the heterogeneity of the meta-analysis results.

In the VAS subgroup analysis of MCHF vs. placebo (Table 2), differences in risk bias and randomization methods may account for the observed variations in therapeutic effects in the MCHF group (P_*interaction*_ < 0.05). However, publication years, sample size and gender ratio were not significant factors influencing the therapeutic effects of MCHF, as no significant interaction was found between their *P*-values (P_*interaction*_ > 0.05).

**TABLE 2:** VAS subgroup analysis of the MCHF and placebo.

Subgroup analyses	Included trials	No. of patients	SMD (95% CI)	P interaction
Overall	5	811	−0.77 [−1.43, −0.12]	
Published year				0.07
Within ten years	2	525	−0.29 [−0.69, 0.12]	
More than 10 years	3	286	−1.06 [−1.80, −0.32]	
Randomization method				<0.00001
SAS	2	525	−0.29 [−0.69, 0.12]	
Random number table method	1	42	−0.19 [−0.75, 0.38]	
Not mentioned	2	238	−1.46 [−1.76, −1.17]	
Sample sizes				0.26
<60	2	94	−0.39 [−0.79, 0.02]	
≧60	3	717	−1.01 [−2.01, −0.01]	
Gender ratio				
More males	3	645	−0.60 [−1.49, 0.28]	0.47
Not mentioned	2	166	−1.05 [−1.88, −0.22]	
Risk bias				
Low risks	3	573	−0.20 (−0.38, −0.01)	<0.00001
Some concerns risk	2	238	−1.46 (−1.76, −1.17)	

In all sensitivity analyses, no changes in statistical significance were observed for any outcome, consistent with the results of the overall analysis (Table 3).

**TABLE 3:** VAS sensitivity analyses of MCHF vs. placebo.

Sensitivity analyses	Excluded trials	Included trials	I^2^(%)	SMD (95% CI)	*P*
Overall analysis		5	92	−0.77 [−1.43, −0.12]	0.02
Excluding small trials (number of participants < 60)	2	3	96	−1.01 [−2.01, −0.01]	0.05
excluding trials with follow-up ≦14 days	0	5	92	−0.77 [−1.43, −0.12]	0.02
Using fixed-effects model	0	5	92	−0.55 [−0.71, −0.39]	0.00
Excluding the largest trial	1	4	84	−0.96 [−1.57, −0.35]	0.02
Excluding the Jingshu Granule group	1	4	84	−0.96 [−1.57, −0.35]	0.02
Excluding the Qishe Pill group	2	3	96	−1.01 [−2.01, −0.01]	0.05
Excluding the Jingtong Granule group	2	3	0	−0.20 [−0.38, −0.01]	0.04
Excluding studies that did not mention randomization methods	2	3	0	−0.20 [−0.38, −0.01]	0.04

##### 3.4.1.2 MCHF plus conventional oral drugs and conventional oral drugs

**FIGURE 7:**
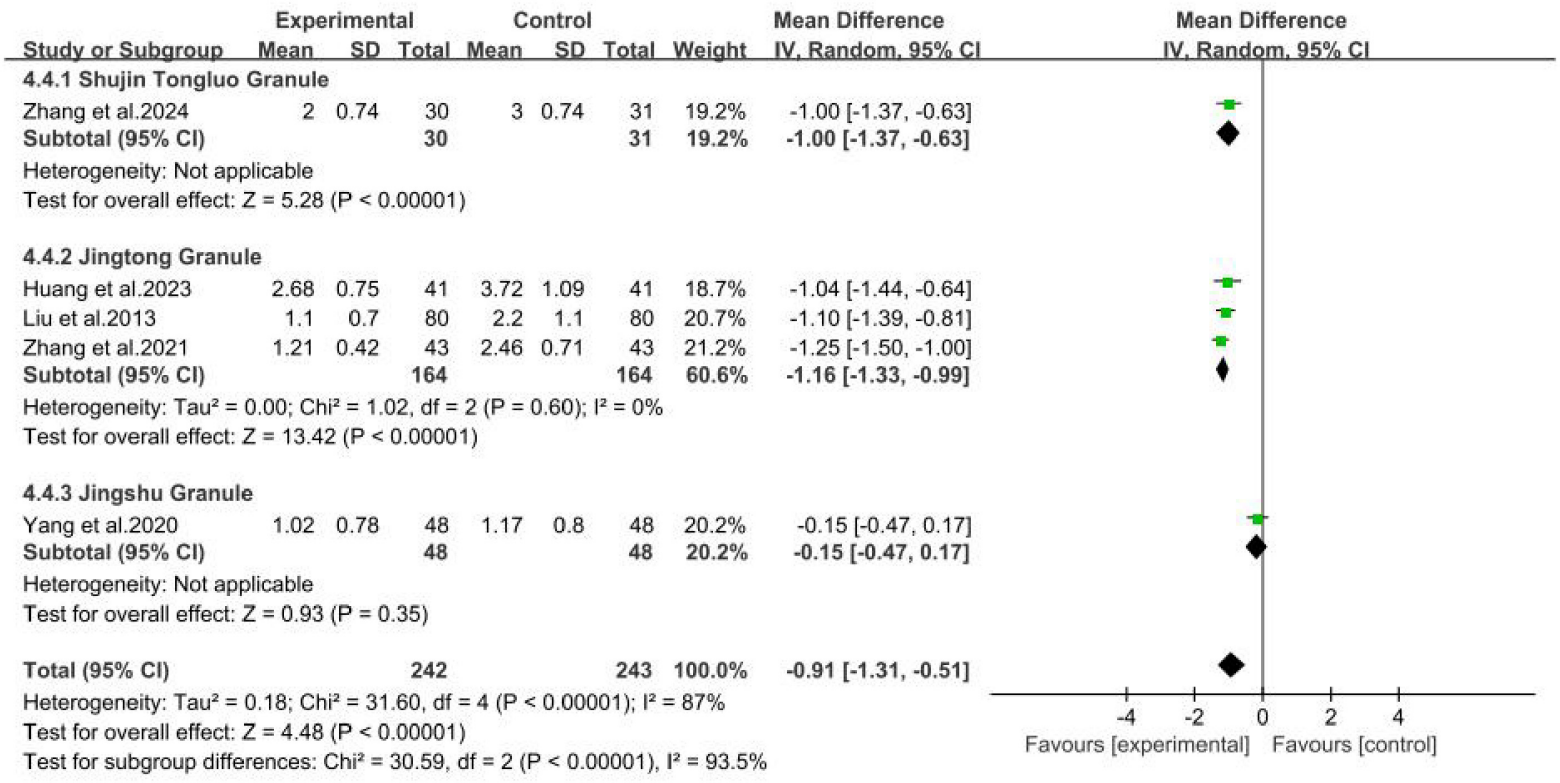
VAS forest plot of MCHF plus conventional oral drugs and conventional oral drugs.

The five included studies (Huang et al., 2023; Liu et al., 2013; Yang et al., 2020; Zhang et al., 2021; Zhang et al., 2024), involving a total of 485 patients, compared the efficacy of MCHF plus conventional oral drugs vs. conventional oral drugs alone (Figure 7). The results indicated that MCHF combined with conventional oral drugs was more efficacious in improving pain.

In our subgroup analyses (Table 4). All subgroup analyses are not possible reasons for the different therapeutic effects of MCHF, and there is no interaction between their *P*-values (P_*interaction*_ > 0.05). The conditions for sensitivity analysis may not be the main cause of heterogeneity.

**TABLE 4:** VAS subgroup analysis of the MCHF plus conventional oral drugs and conventional oral drugs.

Subgroup analyses	Included trials	No. of patients	MD (95% CI)	P interaction
Overall	5	485	−0.91(−1.31, −0.51)	
Published year				
Within 10 years	4	325	−0.86 (−1.38, −0.34)	0.43
More than 10 years	1	160	−1.10(−1.39, −0.81)	
Randomization method				
Random number table method	3	239	−0.72 (−1.32, −0.12)	0.15
Not mentioned	2	246	−1.19 (−1.37, −1.00)	
Gender ratio				
More males	3	338	−0.76 (−1.39, −0.13)	0.24
More females	2	147	−1.17 (−1.38, −0.97)	
Duration of treatment (day)				
≦14	2	246	−1.19 (−1.37, −1.00)	0.15
>14	3	239	−0.72 (−1.32, −0.12)	
Risk bias				
Low risks	0	0	–	–
some concerns risk	5	485	–0.91(−1.31, –0.51)	

For each of our sensitivity analyses (Table 5), the results of all sensitivity analyses showed that there was significant difference between MCHF plus conventional oral drugs and conventional oral drugs. The results of sensitivity analyses were robust.

**TABLE 5:** VAS sensitivity analyses of MCHF plus conventional oral drugs and conventional oral drugs.

Sensitivity analyses	Excluded trials	Included trials	I^2^(%)	MD (95% CI)	*P*
Overall analysis	0	5	87	–0.91(−1.31, –0.51)	
Excluding small trials (number of participants < 60)	0	5	87	–0.91(−1.31, –0.51)	0.00
excluding trials with follow-up ≦14 days	2	3	87.9	–0.72(−1.32, –0.12)	0.02
Using fixed-effects model	0	5	87	–0.94(−1.08, –0.81)	0.00
Excluding the largest trial	1	3	90	–0.86(−1.38, –0.34)	0.001
Excluding the Jingshu Granule group	1	3	0	–1.13 [−-1.29, –0.98]	0.00
Excluding the Shujin Tongluo Granule group	1	3	90	–0.89 [−-1.38, –0.39]	0.00
Excluding the Jingtong Granule group	3	2	91	–0.57 [−-1.40, 0.26]	0.18
Excluding studies that did not mention randomization methods	2	3	88	–0.72 [−-1.32, –0.12]	0.02

#### 3.4.2 MCHF and conventional oral drugs

**FIGURE 8:**
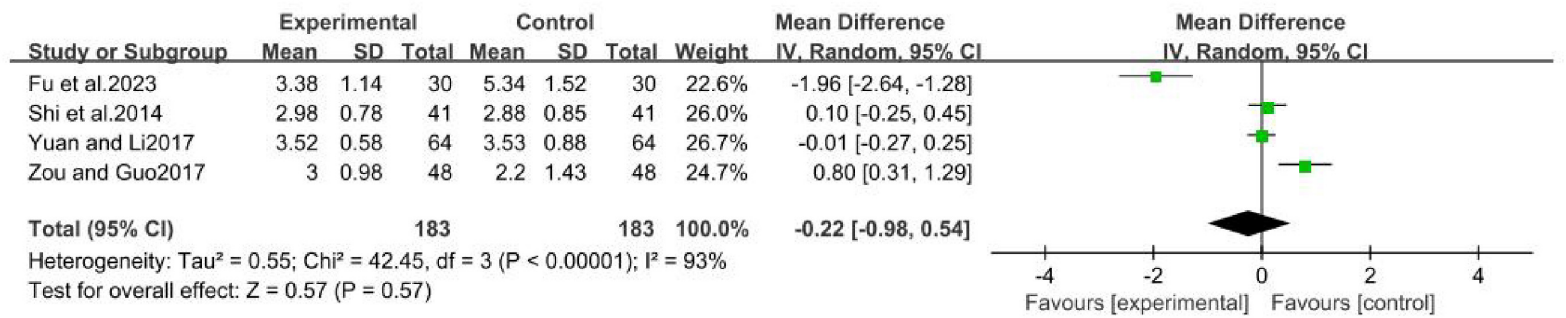
VAS forest plot of MCHF and conventional oral drugs.

The four studies (Fu et al., 2023; Shi et al., 2014; Yuan and Li, 2017; Zou and Guo, 2017), involving 366 patients, directly compared the efficacy of MCHF with conventional oral drugs (Figure 8). The results indicated that there was no statistically significant difference between the efficacy of MCHF and conventional oral drugs (MD = -0.22, 95% CI: −0.98 to −0.54, *P* = 0.57 > 0.05; *I*^2^ = 93%). However, since the four studies in this group utilized different MCHF, a meta-analysis was not performed for subgroup analysis.

In the VAS subgroup analysis of MCHF vs. conventional oral drugs (Table 6), differences in publication years and randomization methods may account for the observed variations in therapeutic effects in the MCHF group (P_*interaction*_ < 0.05). However, the duration of treatment and gender ratio were not significant factors influencing the therapeutic effects of MCHF, as no significant interaction was found between their subgroup *P*-values (P_*interaction*_ > 0.05).

**TABLE 6:** VAS subgroup analysis of the MCHF and conventional oral drugs.

Subgroup analyses	Included trials	No. of patients	I^2^(%)	MD (95% CI)	P interaction
Overall	4	366	93	–0.22(−0.98, –0.54)	
Published year					0.008
Within ten years	3	284	95	–0.36 (−1.56, 0.85)	
More than 10 years	1	82		−0.1(−0.25, −0.45)	
Randomization method					0.01
SAS	1	82		−0.1(−0.25, −0.45)	
Random number table method	2	188	96	−0.96 (−2.87, 0.95)	
Not mentioned	1	96		0.8 (0.31, 1.29)	
Gender ratio					
More males	2	156	98	−0.57 (−3.27, 2.14)	0.18
More females	2	110	0	0.03 (−0.18, 0.24)	
Duration of treatment (day)					
≦14	2	188	96	−0.96 (−2.87, 0.95)	0.25
>14	2	178	81	0.43 (−0.26, 1.11)	
Risk bias					
Low risks	0	0	–	–	–
Some concerns risk	4	366	93	−0.22(−0.98, −0.54)	

The results of all sensitivity analyses indicated that there was no significant difference between MCHF and conventional oral drugs. These findings are consistent with the results of the overall analysis (Table 7).

**TABLE 7:** VAS sensitivity analyses of MCHF and conventional oral drugs.

Sensitivity analyses	Excluded trials	Included trials	I^2^(%)	MD (95% CI)	*P*
Overall analysis	0	4	93	−0.22(−0.98, −0.54)	0.57
Excluding small trials (number of participants < 60)	0	4	93	−0.22(−0.98, −0.54)	0.57
excluding trials with follow-up ≦14 days	2	3	87.8	−0.37(−0.42, −1.16)	0.36
Using fixed-effects model	0	5	93	−0.01(−0.19, −0.18)	0.92
Excluding the largest trial	1	3	95	−0.33(−1.64, −0.98)	0.62
Excluding the Jingfukang Granule	1	3	76	0.25 [−0.17, 0.67]	0.24
Excluding the Biqi Capsule	1	3	95	−0.33 [−1.64, 0.98]	0.62
Excluding the Tenghuang Jiangu Tablet	1	3	93	−0.56 [−1.43, 0.32]	0.21
Excluding the Gentongping Granule	1	3	95	−0.36 [−1.56, 0.85]	0.56
Excluding studies that did not mention randomization methods	1	3	93	−0.56 [−1.43, 0.32]	0.21

### 3.5 NDI-meta

#### 3.5.1 MCHF vs. placebo and MCHF plus conventional oral drugs vs. conventional oral drugs

**FIGURE 9:**
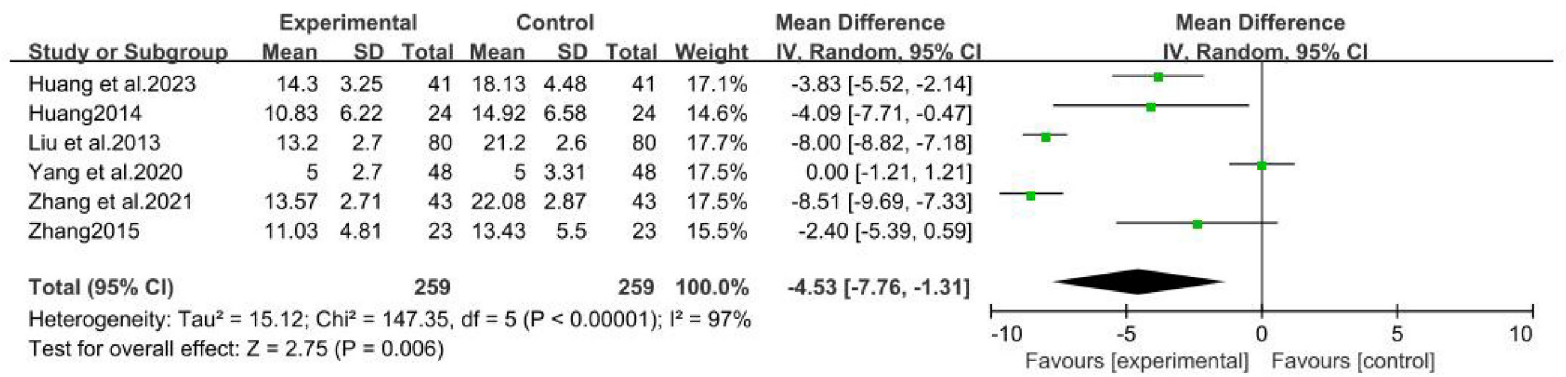
NDI forest plot of MCHF plus conventional drugs vs. conventional drugs alone and MCHF vs. placebo.

Six studies (Huang et al., 2023; Huang, 2014; Liu et al., 2013; Yang et al., 2020; Zhang et al., 2021; Zhang, 2015), involving 518 patients, compared the Neck Disability Index (NDI) of MCHF plus conventional oral drugs vs. conventional oral drugs alone, and MCHF vs. placebo (Figure 9). The results indicated that the therapeutic effect of the MCHF group was superior to that of the control group (MD = -4.35, 95% CI: −7.76 to −1.13, *P* < 0.05; *I*^2^ = 97%).

**FIGURE 10:**
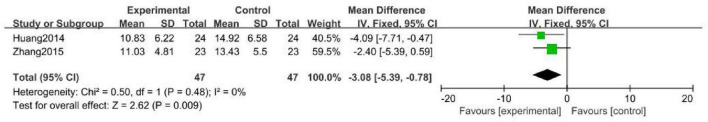
NDI forest plot of MCHF and placebo.

In the two studies (Huang, 2014; Zhang, 2015) that compared MCHF with placebo separately (Figure 10), the results showed that MCHF was superior to placebo after treatment (WMD = -3.08, 95% CI: −5.39 to −0.78, *P* < 0.05; *I*^2^ = 0%).

**FIGURE 11:**
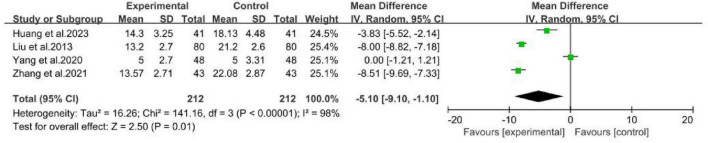
NDI forest plot of MCHF plus conventional drugs vs. conventional drugs alone.

The four studies (Huang et al., 2023; Liu et al., 2013; Yang et al., 2020; Zhang et al., 2021) compared MCHF combined with conventional oral drugs to conventional oral drugs alone as the control group (Figure 11). The results indicated that the NDI score in the MCHF group was superior to that of the control group after treatment (WMD = -5.1, 95% CI: −9.1 to −1.1, *P* < 0.05; *I*^2^= 98%).

#### 3.5.2 MCHF and conventional oral drugs

Only one study (Fu et al., 2023) compared MCHF with conventional oral drugs (MCHF: 13.42 ± 2.73, conventional oral drugs: 20.61 ± 3.57), therefore, a meta-analysis was not conducted.

### 3.6 Adverse events

Nine studies reported the occurrence or absence of adverse events, while five studies did not provide data on adverse events. One study reported no adverse events in either the MCHF group or the control group. The reported adverse events included gastrointestinal symptoms, abnormal liver function, dizziness, rashes, and neurological symptoms. The majority of adverse events were mild and resolved spontaneously after discontinuation of the drug or with symptomatic treatment. No serious adverse events were reported.

#### 3.6.1 MCHF vs. placebo and MCHF plus conventional oral drugs vs. conventional oral drugs

**FIGURE 12:**
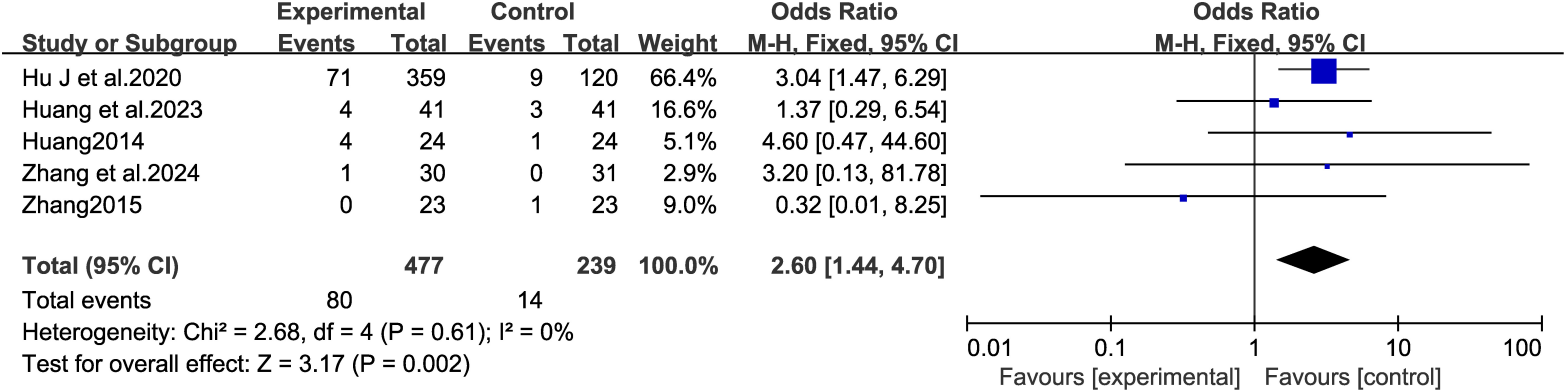
Adverse Events forest plot of MCHF plus conventional drugs vs. conventional drugs alone and MCHF vs. placebo.

The five studies (Hu et al., 2020; Huang et al., 2023; Huang, 2014; Zhang et al., 2024; Zhang, 2015), involving 716 patients, compared MCHF vs. placebo and MCHF plus conventional oral drugs vs. conventional oral drugs (Figure 12). The results indicated that the incidence of adverse events in the MCHF group was higher than that in the control group after treatment (OR = 2.6, 95% CI: 1.44–4.7, *P* < 0.05; *I*^2^ = 0%). For rare events or zero-cell study events, the Peto OR method was employed to re-pool the effect sizes. The results demonstrated that the direction of the effect derived from the Peto OR was completely consistent with that of the original Mantel-Haenszel method. Although the 95% CI was slightly narrowed, the statistical significance of the original conclusion remained unchanged (Peto OR = 2.28, 95% CI: 1.39–3.74). We also applied the continuity correction method with the addition of 0.5 for effect size pooling when dealing with data containing zero cells. After correction, the effect size values showed no substantial differences from those of the original analysis, and none of the corrected confidence intervals (CIs) crossed the line of no effect, which further verified the stability of the results (OR = 2.29, 95% CI: 1.25–4.21).

**FIGURE 13:**
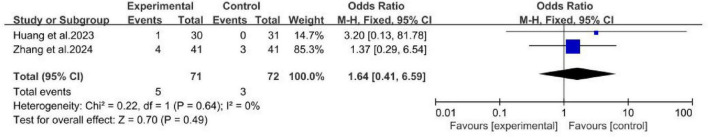
Adverse Events forest plot of MCHF plus conventional oral drugs vs. conventional oral drugs.

**FIGURE 14:**
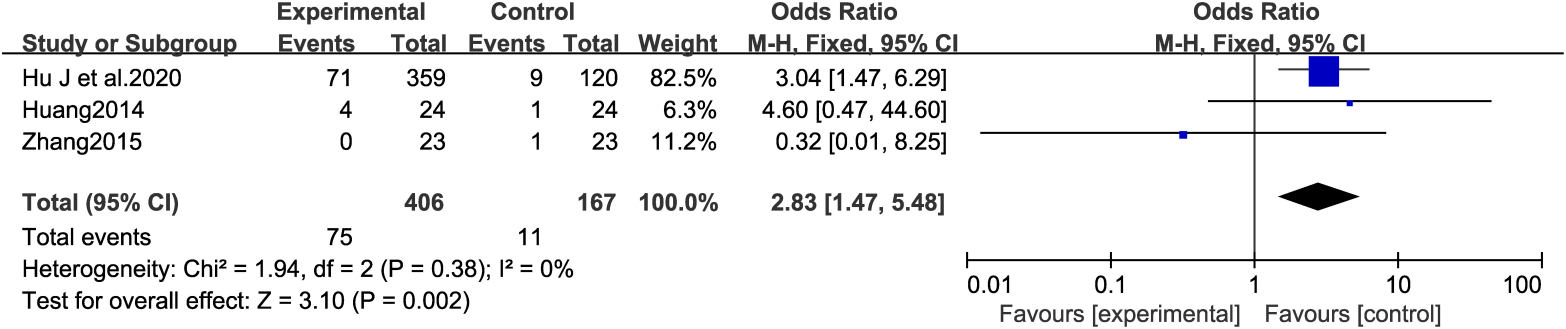
Adverse Events forest plot of MCHF vs. placebo.

A meta-analysis was conducted to compare the incidence of adverse events between the MCHF group and the control group. No significant difference was found in the incidence of adverse events between the MCHF plus conventional oral drugs and conventional oral drugs treatment groups (Higgins et al., 2024; Myung, 2023) (OR = 1.64 [0.41, 6.59]; *P* = 0.486; *I*^2^ = 0.0%) (Figure 13). However, the comparison between MCHF and placebo treatment groups suggested that the frequency of adverse events was higher in the MCHF group (OR = 2.83 [1.47, 5.48]; *P* < 0.05; *I*^2^ = 0.0%) (Figure 14).

#### 3.6.2 MCHF vs. conventional oral drugs

**FIGURE 15:**
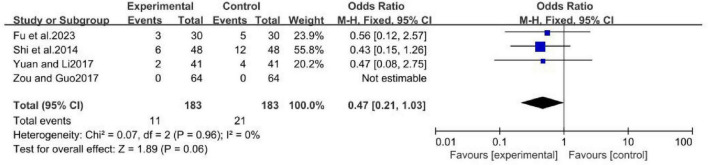
Adverse Events forest plot of MCHF and conventional oral drugs.

A fixed-effect model was used in the meta-analysis of MCHF and conventional oral drugs treatment groups (Fu et al., 2023; Shi et al., 2014; Yuan and Li, 2017; Zou and Guo, 2017), which included a total of 366 patients. The results indicated that there was no significant difference in the incidence of adverse events between the MCHF and conventional oral drug treatment groups (OR = 0.47 [0.21, 1.03]; *P* = 0.06; *I*^2^ = 0%) (Figure 15). For rare events or zero-cell study events, the Peto OR method was employed to re-pool the effect sizes. The results demonstrated that the direction of the effect derived from the Peto OR was completely consistent with that of the original Mantel-Haenszel method. Although the 95% CI was slightly changes, the statistical significance of the original conclusion remained unchanged (Peto OR = 0.48, 95% CI: 0.23–1.02). We also applied the continuity correction method with the addition of 0.5 for effect size pooling when dealing with data containing zero cells. After correction, the effect size values showed no substantial differences from those of the original analysis, and none of the corrected confidence intervals (CIs) crossed the line of no effect, which further verified the stability of the results (OR = 0.52, 95% CI: 0.24–1.14).

### 3.7 Grading of evidence level

In our meta-analysis, we assessed the overall quality of existing evidence using the Gradepro Guideline Development Tool (GDT). For the results comparing MCHF plus conventional oral drugs vs. conventional oral drugs or MCHF vs. placebo, one study group (adverse events) was rated as moderate, while two study groups (VAS, NDI) were rated as very low. When comparing MCHF and placebo, one study group (adverse events) was rated as moderate, and three study groups [VAS (all groups), VAS (Qishe group), VAS (Jingtong group), and NDI] were rated as very low. For the comparison between MCHF and conventional oral drugs, one study group (VAS) was rated as very low, and another study group (adverse events) was rated as low. For the results comparing MCHF combined with conventional oral drugs vs. conventional oral drugs, three study groups (VAS, NDI, and adverse events) were rated as very low. These ratings were based on a rigorous evaluation of three factors contributing to “decreased evidence quality” (risk of bias, inconsistency, indirectness, imprecision, and publication bias) and three factors contributing to “improved evidence quality” (large effect, reasonable confounding that could change the effect, and dose-response effect). A summary of the strength of the evidence for the outcomes is presented in Table 8.

**TABLE 8:** Quality of evidence.

Certainty assessment							Summary of findings			
Participants (studies)	Risk of bias	Inconsistency	Indirectness	Imprecision	Publication bias	Overall certainty of evidence	Study event rates (%)		Effect	
							Experimental group	Control group	Relative effect (95% CI)	Absolute
**MCHF plus conventional oral drugs vs. conventional oral drugs or MCHF vs. placebo**										
**VAS**										
10(RCTs)	Serious 1	Very serious 2	No serious	No serious	None	⊕○○○ Very low	791	505	-	SMD 0.97 lower (1.41 to 0.53 lower)
**NDI**										
6(RCTs)	Serious 1	Very serious 2	No serious	No serious	None	⊕○○○ Very low	259	259	-	MD 4.53 lower (7. 76 to 1.31 lower)
**Adverse events**										
5(RCTs)	Serious 1	No serious	No serious	No serious	None	⊕⊕⊕○ Moderate	80/477 (16.8%)	14/239 (6.6%)	OR 2.6 (1.44 to 4.7)	81 more per 1000 (from 24 more to 168 more)
								4.40%		63 more per 1000 (from 18 more to 134 more)
**MCHF compared to placebo for treating cervical radiculopathy**										
**VAS (All group)**										
5(RCTs)	Serious 1	Very serious 2	No serious	Serious 3	None	⊕○○○ Very low	549	262	-	SMD 0.77 lower (1.43 to 0.12 lower)
**VAS (Qishe group)**										
2(RCTs)	Serious 1	No serious	No serious	Very serious 4	None	⊕○○○ Very low	47	47	-	SMD 0.39 lower(0. 79 to 0.02 higher)
**VAS (Jingtong group)**										
2(RCTs)	Serious 1	No serious	No serious	Serious 5	None	⊕⊕○○ Low	143	95	-	SMD 1.46 lower (1. 76 to 1.17 lower)
**NDI**										
2(RCTs)	Serious 1	No serious	No serious	Very serious 4	None	⊕○○○ Very low	47	47	-	MD 3.08 lower (5.39 to 0.78 lower)
**Adverse events**										
3(RCTs)	Serious 1	No serious	No serious	No serious	None	⊕⊕⊕○ Moderate	75/406 (18.5%)	11/167 (6.6%)	OR 2.83 (1.47 to 5.48)	100 more per 1000 (from 28 more to 213 more)
								4.40%		71 more per 1000 (from 19 more to 157 more)
**MCHF compared to conventional oral drugs alone for treating cervical radiculopathy**										
**VAS**										
5(RCTs)	Serious 1	Very serious 2	No serious	Very serious 4	None	⊕○○○ Very low	183	183		MD 0.22 lower (0.98 to 0.54 higher)
**Adverse events**										
4(RCTs)	Serious 1	Very serious 2	No serious	Serious 3	None	⊕⊕○○ Low	11/183 (6%)	21/183 (11.5%)	OR 0.47 (0.21 to 1.03)	57 fewer per 1000 (from 88 fewer to 3 more)
								13.20%		65 fewer per 1000 (from 101 fewer to 3 more)
**MCHF combined with conventional oral drugs compared to conventional oral drugs for treating cervical radiculopathy**										
**VAS**										
5(RCTs)	Serious 1	Very serious 2	No serious	Serious 3	None	⊕○○○ Very low	242	243		MD 0.91 lower (1.31 to 0.51 lower)
**NDI**										
4(RCTs)	Serious 1	Very serious 2	No serious	No serious	None	⊕○○○ Very low	212	212		MD 5.1 lower (9.1 to 1.1 lower)
**Adverse events**										
2(RCTs)	Serious 1	No serious	No serious	Very serious 6	None	⊕○○○ Very low	5/171 (7%)	3/72 (4.2%)	OR 1.64 (0.41 to 6.59)	25 more per 1000 (from 24 fewer to 181 more)
								3.70%		22 more per 1000 (from 21 fewer to 165 more)

1: All studies were assessed to be medium risk of bias. 2: I^2^≥ 75%. 3: 95%CI cross equivalence line. 4: Sample size less than 400 and 95%CI cross equivalence line. 5: Sample size less than 400. 6: Sample size less than 300 and 95%CI cross equivalence line.

### 3.8 Trial sequential analysis

**FIGURE 16:**
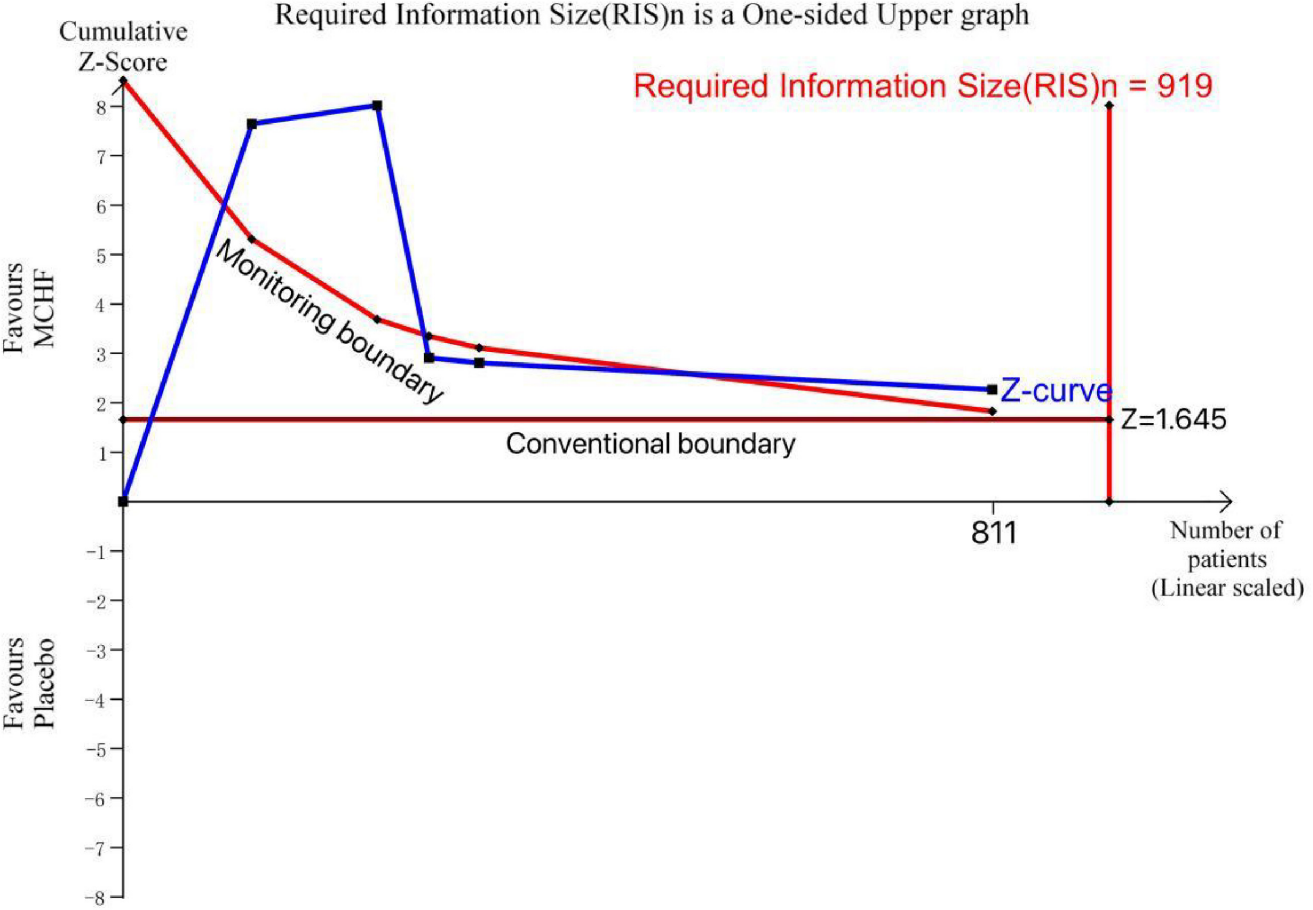
TSA plot of MCHF vs. placebo.

We performed TSA for the primary outcome, VAS (MCHF vs. placebo), using data from 5 RCTs involving 811 patients. A superiority comparison was conducted using a one-sided upper graph (Figure 16).

The cumulative Z-curve has effectively crossed the conventional and monitoring boundary, it can be stated that the observed effect is significant and there is very little chance of Type-I error, and future studies’ data are unlikely to show any variable effect. However, the cumulative Z-curve did not meet the predetermined required information size (RIS) of 919. Although these findings demonstrated marginal statistical significance, further clinical trials are still required to verify that the efficacy of MCHF in the treatment of CR was superior to that of placebo.

**FIGURE 17:**
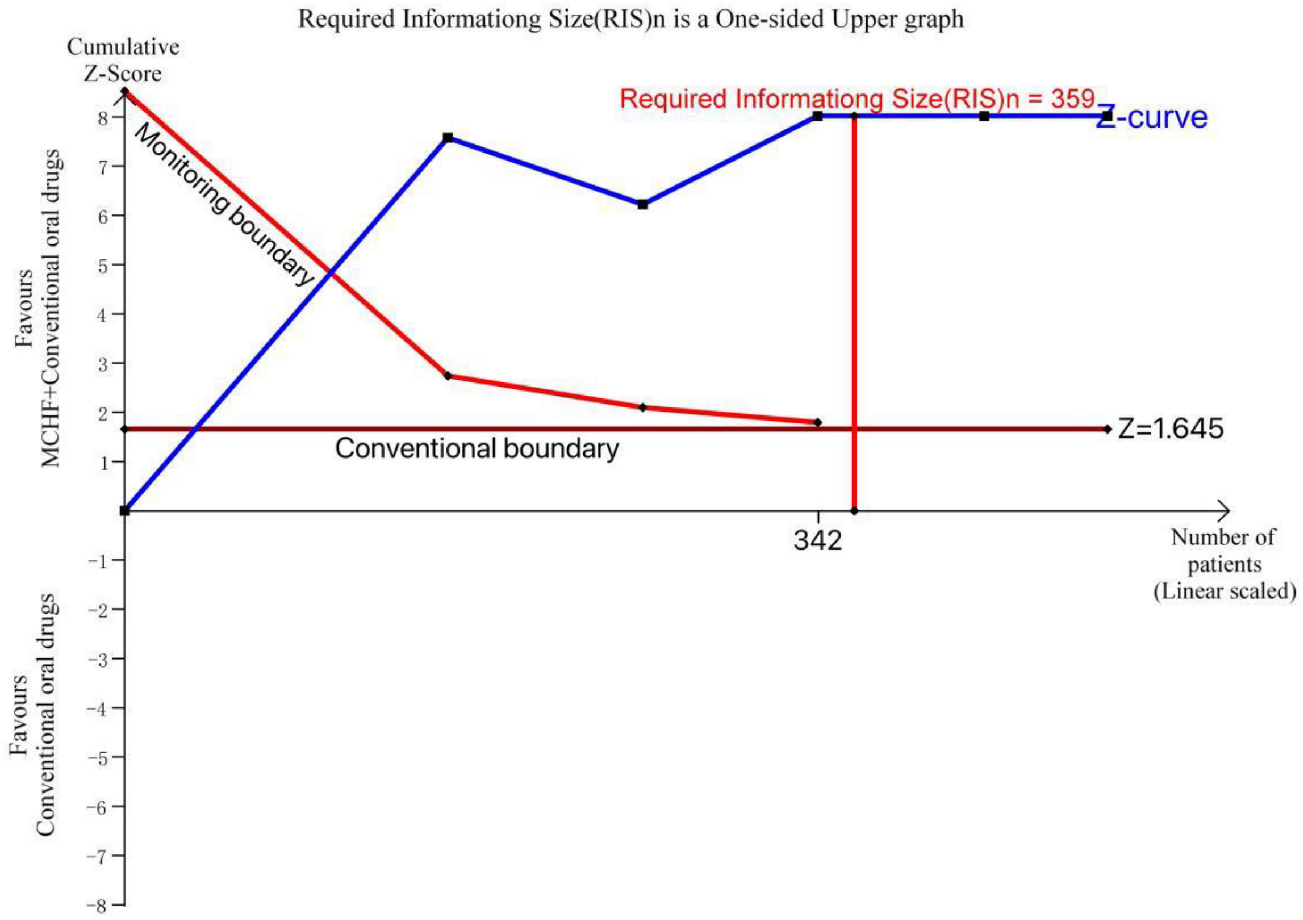
TSA plot of MCHF plus conventional oral drugs vs. conventional oral drugs.

For 485 patients in 5 RCTs comparing VAS (MCHF plus conventional oral drugs vs. conventional oral drugs), TSA indicated that the cumulative Z-curve intersected both the conventional boundaries, the monitoring boundary, and the RIS (359) in a one-sided upper graph. TSA showed that the accumulated evidence was sufficient to verify the robustness on the efficacy of MCHF plus conventional oral drugs in CR compared to conventional oral drugs (Figure 17).

**FIGURE 18:**
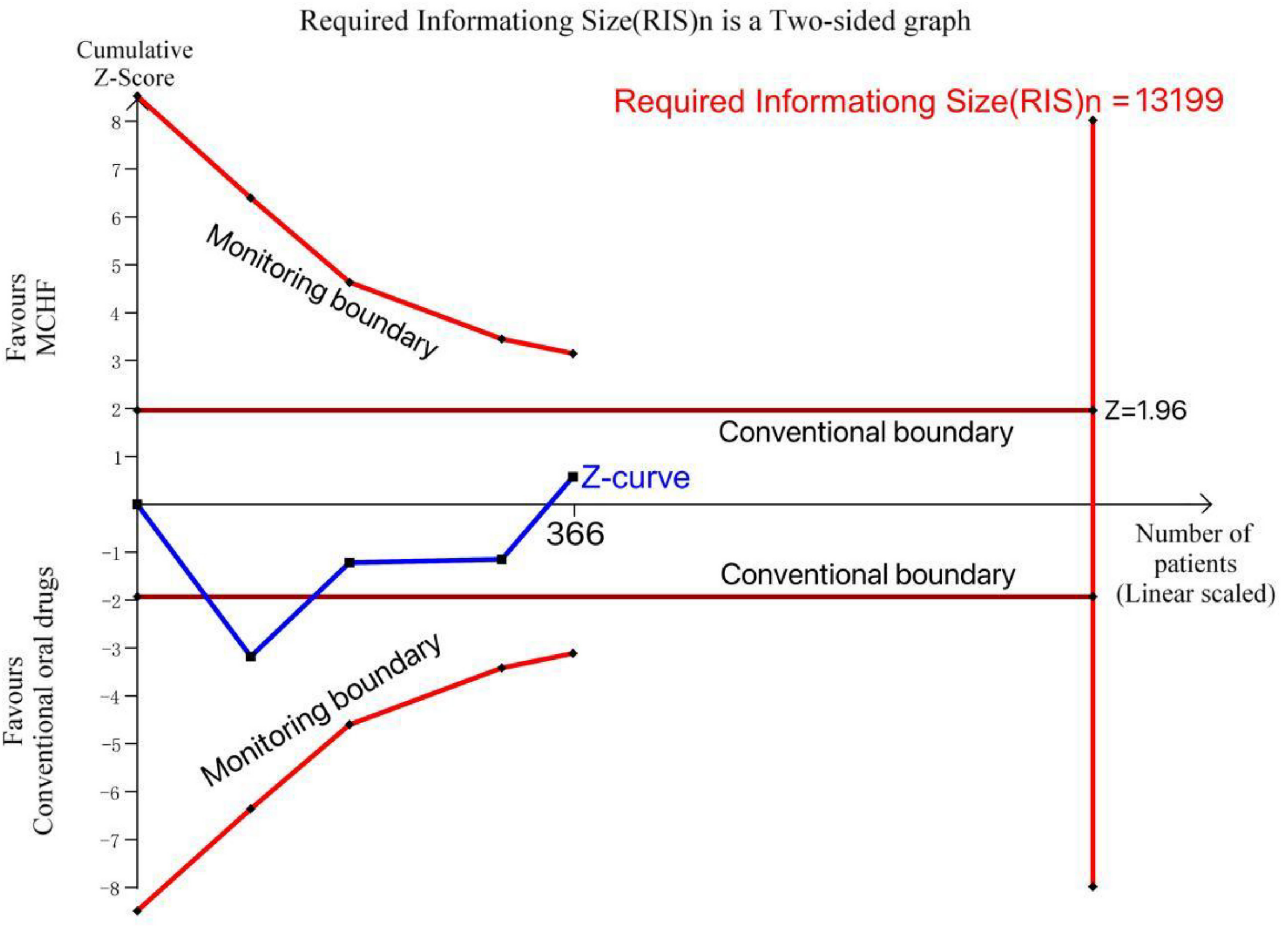
TSA plot of MCHF vs. conventional oral drugs.

We performed a difference comparison and two-sided upper graph for VAS (MCHF vs. conventional oral drugs) using data from 5 RCTs involving 485 patients. The cumulative Z-curve did not reach the conventional boundaries or monitoring boundary or the RIS (13,199). This suggests that additional RCTs are required to verify the results in the study of MCHF vs. conventional oral drugs (Figure 18).

## 4 Discussion

CR is a condition caused by compression or damage to the nerve roots at the base of the cervical spine. It is typically characterized by symptoms such as pain, numbness, and weakness in the neck, shoulders, and upper limbs (Woods and Hilibrand, 2015). In severe cases, CR can lead to sensory loss and motor dysfunction, significantly impacting the patient’s daily activities and work.

MCHF have been applied in the treatment of various orthopedic diseases, including knee osteoarthritis, osteoporosis, and lumbar disc herniations (Li et al., 2021; Zhang et al., 2022; Zhu et al., 2015b). These studies demonstrate that MCHF holds significant potential and clinical value in the treatment of orthopedic-related conditions. Relevant research suggests that the efficacy of MCHF may be attributed to its multi-component, multi-target mechanisms. These herbal components may alleviate the symptoms of cervical radiculopathy (CR) by regulating immune responses, reducing local inflammation, and improving neurovascular supply (Duan et al., 2025; Niu et al., 2024).

Although several studies have investigated the use of MCHF in the treatment of CR, meta-analyses on MCHF for CR remain relatively scarce. Existing literature has published a meta-analysis on only a single MCHF for treating CR (Zhu et al., 2015a). Given the limited scope of studies focusing on a single MCHF, we conducted an integrated analysis of multiple MCHF formulations, providing a more comprehensive and representative assessment.

In this systematic review and meta-analysis, we included 14 studies, encompassing 8 types of Manufactured Chinese Herbal Formulas (MCHF): Shujin Tongluo Granules, Jingtong Granules, Jingfukang Granules, Jingshu Granules, Biqi Capsules, Tenghuang Jiangu Tablets, Qishe Pill, and Gentongping Granules, with a total of 1,576 participants. We performed a meta-analysis on the primary and secondary outcomes, as well as subgroup analyses. Due to limited data, we did not conduct a meta-analysis for other outcome measures.

The studies were categorized into three types: placebo-controlled studies, add-on studies, and head-to-head studies. Our findings indicate that MCHF may alleviate symptoms of CR, as evidenced by reduced pain and improved function. According to the VAS score analysis, MCHF was found to be comparable to conventional oral drugs in reducing pain. In the meta-analysis of add-on trials and placebo-controlled trials, the intervention group achieved better efficacy than the control group. Analysis of NDI scores demonstrated that the intervention group achieved greater improvements in functional outcomes than the control group in both add-on studies, and head-to-head trials. Given that only one study conducted an NDI comparison between MCHF and conventional oral medications, no meta-analysis was carried out, and the difference in functional improvement between these two approaches could not be established. Regarding adverse events, no significant differences were observed between the two groups in both add-on studies, and head-to-head trials. In contrast, the incidence of adverse events in the intervention group was higher than that in the control group in placebo-controlled trials. MCHF did not cause severe adverse reactions and did not result in significant damage to organ systems. However, 5 out of the 14 included studies failed to report adverse events, indicating the presence of selective reporting of adverse events. The most direct consequence of unreported adverse events is the potential underestimation of the true incidence, severity, or relevance of adverse events. In the context of the present study, this shortcoming significantly weakened the robustness of the conclusions regarding safety. It distorted the actual adverse event profile, artificially reduced the pooled risk, and created a false sense of security. Furthermore, this issue may introduce publication bias, as unpublished data or unreported studies are likely to contain additional safety-related information. This not only compromised the balanced risk-benefit assessment, but also misled clinicians and patients, and ultimately diminished the reliability of evidence graded by the GRADE framework.

The analysis of primary efficacy indicators revealed significant heterogeneity. Subgroup analysis, meta-regression, and sensitivity analysis were performed, but no significant factors influencing the heterogeneity were identified. We believe that the different drug formulations may be the primary source of heterogeneity in the meta-analysis. Other potential factors were not addressed in the original studies. Future clinical research should report more comprehensive study characteristics and details to facilitate the identification of additional factors in subsequent systematic reviews.

The overall quality of the studies was rated as moderate, low, or very low according to the GRADE framework. The presence of low-quality studies may influence the evaluation of efficacy and safety. The lower quality of evidence could be attributed to factors such as randomization methods, blinding procedures, and sample sizes in the included studies. We recommend that future randomized controlled trials (RCTs) adhere strictly to the quality assessment criteria outlined in the Cochrane Handbook to improve the overall quality of the literature.

Trial Sequential Analysis (TSA) was performed to assess the reliability of the meta-analysis results. Although TSA provided sufficient information to confirm the conclusions regarding the efficacy of MCHF relative to placebo in pain relief, as well as the efficacy of MCHF combined with conventional oral drugs vs. conventional oral drugs alone in pain relief, the reliability of these conclusions still requires further validation through additional high-quality RCTs in the future—due to limitations arising from bias, poor reporting quality, and clinical heterogeneity, which the present study was unable to overcome. For the comparison between MCHF and conventional oral drugs, both treatments show comparable efficacy, and there may be a risk of Type II error (false negative results). Therefore, further large-scale RCTs are also required to validate these findings.

Regarding the limitations of this study, most of the included studies had small sample sizes, and the varying compositions of the MCHF formulations contributed to some heterogeneity. Additionally, since all the included studies were conducted in China, more multi-center, randomized controlled trials (RCTs) from other countries and regions are needed in the future to assess the generalizability of these findings. The randomization method is a critical aspect of RCTs; however, over one-third of the included studies did not report the random allocation method. Furthermore, the majority of studies did not disclose the composition or manufacturing process of the placebo. We also did not contact the authors of the studies via phone or email for more detailed information. Due to limitations in the available literature, we were unable to perform a follow-up efficacy analysis for MCHF. As the quantity and quality of the literature improve in the future, follow-up analyses may be conducted. In addition, regarding the meta-analysis results of the present study, although the vast majority of mean differences demonstrated statistical significance, none reached the MCID of 2 points (Chung et al., 2017). Furthermore, most included studies failed to analyze the score differences between baseline and end of study, nor did they use comparisons between pre-post intervention differences and MCID as the basis for drawing study conclusions. Owing to limitations in the most of included primary studies stemming from methodological quality issues, the statistically significant findings for MCHF may not align with real-world clinical outcomes.

## 5 Conclusion

In the treatment of CR, MCHF may alleviate pain and improve cervical spine function to a certain extent, yet it is associated with mild adverse reactions. However, the certainty of evidence is low/very low, with safety evidence being particularly weak. Further high-quality RCTs are warranted to verify the efficacy and safety of MCHF.

## Data Availability

The original contributions presented in the study are included in the article/Supplementary material, further inquiries can be directed to the corresponding authors.

## Footnotes

1
https://www.crd.york.ac.uk/PROSPERO/view/CRD42024605787

2
https://www.clinicaltrials.gov

3
https://www.clinicaltrialsregister.eu

4
https://www.who.int/clinical-trials-registry-platform
