# Supplementary material for: Efficacy and safety of manufactured Chinese herbal formula for cervical radiculopathy: a systematic review with meta-analysis and trial sequential analysis
Source: Front Neurosci. 2026 Feb 10;20:1721569. doi: 10.3389/fnins.2026.1721569 (PMC12929525; doi:10.3389/fnins.2026.1721569)
Supplement: Supplementary file 1 [file Table_1.docx]

**1.The detailed search terms in PubMed were as follows:**

#1.((((((((Medicine, Chinese Traditional[MeSH Terms]) OR (Drugs, Chinese Herbal[MeSH Terms])) OR (Chinese [Title/Abstract]) OR (tablet [Title/Abstract])) OR (pill [Title/Abstract])) OR (powder [Title/Abstract])) OR (capsule [Title/Abstract])) OR (granule [Title/Abstract])) OR (oralliquid [Title/Abstract]))

#2. ((((Radiculopathy [MeSH Terms]) OR (cervical spondylotic radiculopathy [Title/Abstract])) OR (cervical radiculopathy [Title/Abstract])) OR (nerve root type cervical spondylosis [Title/Abstract]))

#3. (((randomized [Text Word] OR random [Text Word]) OR controlled [Text Word]) OR control [Text Word]) OR trial [Text Word]

#4. #1AND#2AND#3 Filters: Clinical trial; Humans.

Limits: September 30. 2024

**2.** **中文数据库中国知网的检索策略如下(The detailed search terms in China National Knowledge Infrastructure database were as follows)**

(((主题= (神经根型颈椎病) 或者 题名= (神经根型颈椎病) 或者 v_subject= (中英文扩展(神经根型颈椎病)) 或者 title= (中英文扩展(神经根型颈椎病)) 并且 (主题= (中成药+中药+颗粒+冲剂+胶囊+口服液+片+丹+丸+散) 或者 题名= (中成药+中药+颗粒+冲剂+胶囊+口服液+片+丹+丸+散) 或者 v_subject= (中英文扩展(中成药)+中英文扩展(中药)+中英文扩展(颗粒)+中英文扩展(冲剂)+中英文扩展(胶囊)+中英文扩展(口服液)+中英文扩展(片)+中英文扩展(丹)+中英文扩展(丸)+中英文扩展(散)) 或者 title= (中英文扩展(中成药)+中英文扩展(中药)+中英文扩展(颗粒)+中英文扩展(冲剂)+中英文扩展(胶囊)+中英文扩展(口服液)+中英文扩展(片)+中英文扩展(丹)+中英文扩展(丸)+中英文扩展(散)))) 并且 (主题= (随机+临床+对照) 或者 题名= (随机+临床+对照) 或者 v_subject= (中英文扩展(随机)+中英文扩展(临床)+中英文扩展(对照)) 或者 title= (中英文扩展(随机)+中英文扩展(临床)+中英文扩展(对照)))) (模糊匹配)

检索截止日期：2024年9月31日
